# Metformin improves diabetic kidney disease

**Published:** 2012-01-01

**Authors:** Seyed Seifollah Beladi Mousavi, Hamid Nasri, Mahmoud Rafieian-Kopaei, Mohamad-Reza Tamadon

**Affiliations:** ^1^Department of Internal Medicine, Ahvaz Jundishapur University of Medical Sciences, Ahvaz, Iran; ^2^Department of Nephrology, Division of Nephropathology, Isfahan University of Medical Sciences, Isfahan, Iran; ^3^Medical Plants Research Center, Shahrekord University of Medical Sciences, Shahrekord, Iran; ^4^Department of Internal Medicine, Semnan University of Medical Sciences, Semnan, Iran

**Keywords:** Metformin, Diabetic nephropathy, Podocyte

**Implication for health policy/practice/research/medical education:**

It is acceptable to interpret that metformin has three different responsibilities including: blood glucose regulatory effect, renal tubular cell protection by acting as an effective antioxidant and finally protective effect on diabetic nephropathy through saving the podocytes. Hence, diabetic patients may benefit from all of these three distinct ameliorative effects.

Diabetes nephropathy is now the major cause of end-stage kidney disease globally in both developed and under progressed countries (1). It is the main diagnosis causing kidney insufficiency in 20–40% of people beginning treatment for end-stage renal disease global (1). Interstitial, vascular and glomerular injuries have been discovered the principal characters of diabetic renal disease (2). It is well found that, apoptosis linked to the development of diabetic nephropathy (2,3). In fact, high glucose augmented apoptosis, a form of programmed cell death presented by cell shrinkage, chromatin condensation and DNA fragmentation, in variety of cell types, especially kidney proximal tubular epithelial cells (2–4). Furthermore, diabetic kidneys are mostly prone to acute tubular damage in various clinical conditions, such as dehydration or post renal obstruction (2–5). It is also well defined that, hyperglycemia, by itself, is an independent risk factor for acute renal tubular injury under these situations. High blood glucose activates the generation of free radicals and oxidative stress in renal tubular cells. Reactive oxygen species (ROS) are believed to be principal mediators for several biologic responses, containing proliferation, extracellular matrix deposition and apoptosis (2–5). Actually, besides of apoptosis of proximal tubular epithelial cells by hyperglycemia, other cell types including endothelial cells and podocytes will also be affected by hyperglycemia (2–5). Thus, one of beneficial treatments in the diabetic nephropathy is using medications for inhibiting or reducing apoptosis to keep kidney tubular cells and podocytes as well. Metformin, an oral anti-diabetic drug in the biguanide class is a commonly prescribed drug to treat high blood glucose in patients with type 2 diabetes mellitus (T2DM) (6). Recent studies have documented that metformin possesses antioxidant properties, too. Reduction of apoptosis, induced by oxidative stress, in endothelial cells and inhibition of vascular dysfunction was also found during metformin treatment (6,7). The beneficial action of this drug is through activation of adenosine monophosphate-activated protein kinase (AMPK). This enzyme plays an important role in protecting cellular functions under energy-restricted conditions. Various studies, confirm that AMPK activation by metformin is secondary to its effect on the mitochondria as the primary target of this agent. There is some proof that the beneficial impact of metformin might be due to its mild inhibition of the mitochondrial respiratory chain, while the critical role of mitochondria in cell death is of significance importance and saving the mitochondria has become a pro-survival cell strategy (6–8). It is possible that the role of mitochondria in programmed cell death is associated with the release of apoptotic signaling molecules. ROS production by mitochondria may also lead significantly to cell injury. Previously, Morales *et al*. found that gentamicin-induced kidney tubular damage is attenuated by metformin (9). It is evident that, ROS play a key role in the toxicity of gentamicin, ensuing in acute renal damage, and gentamicin is a mitochondrial toxin that can imply its toxic properties when excreted by the kidney. Mitochondrial toxicity can also be mediated by ROS as mentioned above too (7–9). ROS is normally produced at low levels by mitochondria, conversely under pathological conditions the intracellular and intra mitochondrial ROS content may be increased. When in certain situations, intracellular ROS content reaches to a toxic level, results in oxidative damage to the mitochondria, causing cell death and malfunctioning of the organ (7–9). To consider the potential kidney protective properties of metformin against gentamicin-induced kidney injury and also finding out whether postpone treatment with metformin in acute renal damage exerts parallel benefits on gentamicin kidney toxicity in rats, we conducted a study on male Wistar rats.

In this study, metformin protected and also improved gentamicin-induced acute kidney damage, thus, this agent might be applicable for protection of renal tubular cells (10). Likewise, Denamur *et al.* (11) observed that co-administration of metformin and gentamicin for 13 days effectively reversed gentamicin-induced renal damage. Thus, these findings provide further proof for the attribution of metformin in its renal protective effectiveness beyond its well-known blood sugar regulatory action (6–10). Accordingly, Kim *et al*. conducted a study using metformin for diabetic rats. They found the repair of podocytes by metformin in diabetic rats. They proposed that diabetes-induced podocyte loss in diabetic kidney disease could be reduced by metformin (12). Kim *et al*, also observed that the phosphorylation of AMPK was decreased in the kidney of diabetic rats, and metformin could return its alteration. Therefore, metformin might exert some of its effects by correction of kidney oxidative stress (12). Thus one might suggest metformin to inhibit the advanced glycation end-products and improve the free-radical defense system, hence, preventing the diabetic renal injury.

It is well known that the damage to the podocytes lead to the occurrence of proteinuria (11,12). Therefore, the loss of glomerular podocytes precedes and predicts the onset of diabetic kidney disease and may be an early pathological presentation of diabetic nephropathy. Metformin significantly decreased albuminuria in patients with T2DM (3–7). Previous studies have also shown the favorable effects of metformin on reduction of macrovascular morbidity and mortality, suggesting anti-atherogenic, antioxidant and anti-inflammatory effects (3–7). Additionally, metformin significantly decreased albuminuria in patients with T2DM, too (3–9). Therefore, it is acceptable to interpret that metformin has three different responsibilities, including: blood glucose regulatory effect, renal tubular cell protection by acting as an effective antioxidant and finally protective effect on diabetic nephropathy through saving the podocytes. Hence, diabetic patients may benefit from all of these three distinct ameliorative effects.

## Authors’ contributions

All authors contributed to the paper equally.

## Conflict of interests

The authors declared no competing interests.

## Ethical considerations

Ethical issues (including plagiarism, misconduct, data fabrication, falsification, double publication or submission, redundancy) have been completely observed by the authors.

## Funding/Support

None declared.

## References

[1] Alebiosu CO, Ayodele OE (2005). The global burden of chronic kidney disease and the way forward. Ethn Dis.

[2] Chao LK, Chang WT, Shih YW, Huang JS (2010). Cinnamaldehyde impairs high glucose-induced hypertrophy in renal interstitial fibroblasts. Toxicol Appl Pharmacol.

[3] Thorp ML (2005). Diabetic nephropathy: common questions. Am Fam Physician.

[4] Yamagishi S, Fukami K, Ueda S, Okuda S (2007). Molecular mechanisms of diabetic nephropathy and its therapeutic intervention. Curr Drug Targets.

[5] Forbes JM, Fukami K, Cooper ME (2007). Diabetic nephropathy: where hemodynamics meets metabolism. Exp Clin Endocrinol Diabetes.

[6] Baradari AG, Emami Zeydi A, Aarabi M, Ghafari R (2011). Metformin as an adjunct to insulin for glycemic control in patients with type 2 diabetes after CABG surgery: a randomized double blind clinical trial. Pak J Biol Sci.

[7] Zorov DB (2010). Amelioration of aminoglycoside nephrotoxicity requires protection of renal mitochondria. Kidney Int.

[8] Sung JY, Choi HC (2012). Metformin-induced AMP-activated protein kinase activation regulates phenylephrine-mediated contraction of rat aorta. Biochem Biophys Res Commun.

[9] Morales AI, Detaille D, Prieto M, Puente A, Briones E, Arevalo M (2010). Metformin prevents experimental gentamicin-induced nephropathy by a mitochondria-dependent pathway. Kidney Int.

[10] Amini FG, Rafieian-Kopaei M, Nematbakhsh M, Baradaran A, Nasri H (2012). Ameliorative effects of metformin on renal histologic and biochemical alterations of gentamicin-induced renal toxicity in Wistar rats. J Res Med Sci.

[11] Denamur S, Tyteca D, Marchand-Brynaert J, Van Bambeke F, Tulkens PM, Courtoy PJ (2011). Role of oxidative stress in lysosomal membrane permeabilization and apoptosis induced by gentamicin, an aminoglycoside antibiotic. Free Radic Biol Med.

[12] Kim J, Shon E, Kim CS, Kim JS (2012). Renal podocyte injury in a rat model of type 2 diabetes is prevented by metformin. Exp Diabetes Res.

